# Administration of Tamoxifen Can Regulate Changes in Gene Expression during the Acute Phase of Traumatic Spinal Cord Injury

**DOI:** 10.3390/cimb45090472

**Published:** 2023-09-13

**Authors:** Eibar E. Cabrera-Aldana, Yalbi I. Balderas-Martínez, Rafael Velázquez-Cruz, Luis B. Tovar-y-Romo, Rosalba Sevilla-Montoya, Angelina Martínez-Cruz, Claudia Martinez-Cordero, Margarita Valdés-Flores, Monica Santamaria-Olmedo, Alberto Hidalgo-Bravo, Gabriel Guízar-Sahagún

**Affiliations:** 1 Department of Genomics Medicine, National Institute of Rehabilitation (INR), Calzada Mexico-Xochimilco 289, Arenal de Guadalupe, Mexico City 14389, Mexico; dreibarcabrera@yahoo.com.mx (E.E.C.-A.); mvaldes@yahoo.com (M.V.-F.); mgso82@hotmail.com (M.S.-O.); 2 Laboratorio de Biología Computacional, Instituto Nacional de Enfermedades Respiratorias, Ismael Cosío Villegas, Calz. de Tlalpan 4502, Belisario Domínguez Secc 16, Tlalpan, Mexico City 14080, Mexico; yalbibalderas@gmail.com; 3 Genomics of Bone Metabolism Laboratory, National Institute of Genomic Medicine (INMEGEN), Periférico Sur 4809, Arenal Tepepan, Mexico City 14610, Mexico; rvelazquez@inmegen.gob.mx; 4 Department of Molecular Neuropathology, Instituto de Fisiología Celular, Universidad Nacional Autónoma de México, Ciudad Universitaria, Circuito Exterior s/n, Mexico City 04510, Mexico; ltovar@ifc.unam.mx; 5 Reproductive Research and Perinatal Health Department, National Institute of Perinatology, Montes Urales 800, Lomas de Virreyes, Mexico City 11000, Mexico; rosalbasevilla@hotmail.com; 6 Department of Experimental Surgery, Proyecto Camina, A.C. 4430 Calz. Tlalpan, Mexico City 14050, Mexico; markim_00@yahoo.com.mx; 7 Regional Hospital of High Specialty of the Bajio, Blvd. Milenio 130, Col. San Carlos la Roncha, León 37660, Guanajuato, Mexico; claudiamartinezcordero@hotmail.com; 8 Research Unit for Neurological Diseases, Instituto Mexicano del Seguro Social, 330 Avenida Cuauhtémoc, Mexico City 06720, Mexico

**Keywords:** spinal cord injury, transcriptome, tamoxifen, gene expression, inflammatory response

## Abstract

Traumatic spinal cord injury (SCI) causes irreversible damage leading to incapacity. Molecular mechanisms underlying SCI damage are not fully understood, preventing the development of novel therapies. Tamoxifen (TMX) has emerged as a promising therapy. Our aim was to identify transcriptome changes in the acute phase of SCI and the effect of Tamoxifen on those changes in a rat model of SCI. Four groups were considered: (1) Non-injured without TMX (Sham/TMX-), (2) Non-injured with TMX (Sham/TMX+), (3) injured without TMX (SCI/TMX-), and (4) injured with TMX (SCI/TMX+). Tamoxifen was administered intraperitoneally 30 min after injury, and spinal cord tissues were collected 24 h after injury. Clariom S Assays Array was used for transcriptome analysis. After comparing Sham/TMX- versus SCI/TMX-, 708 genes showed differential expression. The enriched pathways were the SCI pathway and pathways related to the inflammatory response. When comparing SCI/TMX- versus SCI/TMX+, only 30 genes showed differential expression, with no pathways enriched. Our results showed differential expression of genes related to the inflammatory response after SCI, and Tamoxifen seems to regulate gene expression changes in *Ccr2* and *Mmp12*. Our study contributes data regarding the potential value of tamoxifen as a therapeutic resource for traumatic SCI during the acute phase.

## 1. Introduction

Traumatic spinal cord injury (SCI) causes irreversible tissue damage, generating permanent motor, sensory, and autonomic system impairment [1]. SCI is primarily caused by traffic accidents [2] and it remains one of the most challenging and demanding conditions to manage [3]. To date, pharmacological therapies remain controversial [4]. SCI has a complex pathophysiology that is not fully understood, preventing the development of more effective therapeutic strategies. During the acute phase of the injury, the primary lesions include traumatic physical wounding, breakdown of the vasculature, disruption of the blood–spinal cord barrier, and acute reaction with resident immune cells. Next, secondary lesions can be observed as demyelination, impaired neurotransmission, immune cell infiltration, neuronal apoptosis, and glial scar formation [5]. Therefore, the strategic medical management of SCI has involved the use of drugs that mitigate the acute phase’s secondary injury and minimize neuronal damage [6] caused by molecules such as cytokines and chemokines, which can have a toxic effect on neurons [7]. Drugs approved for the treatment of neuroinflammation include glucocorticoids such as methylprednisolone (MP). However, MP has side effects and it remains an option under current research [8]. 

Emerging evidence suggests differences in neurologic recovery after SCI between sexes [9] and in the neuroprotection that occurs after trauma [10]. These effects seem to be related to sex hormones. Recent studies in animal models of CNS trauma and stroke have demonstrated a beneficial effect induced by estrogens, progesterone, and human chorionic gonadotropin [11,12,13,14]. Nevertheless, adverse effects associated with hormone therapy have also been reported [15]. However, it has been shown that molecules binding to estrogen receptors can have neuroprotective functions, similar to those of estrogens but without adverse effects. Tamoxifen (TMX) is a nonsteroidal selective estrogen receptor modulator that has recently been found to induce neuroprotection in experimental SCI models. Previous studies in rodent models of SCI have shown that TMX can improve motor function; nevertheless, the molecular bases at the gene expression level of this positive effect remain unexplored [16,17,18]. The molecular mechanisms underlying the neuroprotective effect of TMX are not fully elucidated. The effects of TMX have been divided into two broad categories, genomic and non-genomic. Genomic effects are related to the binding of TMX to the estrogen receptors (ERs) and subsequent transcription of key genes, e.g., VEGF transcription seems to be triggered by ERs favoring blood supply at the injured zone [19]. The non-genomic effect may or may not involve the ERs, e.g., TMX can interfere with the MAPK signaling in an ERs-independent fashion [20].

The aim of this study was to identify the transcriptome changes occurring in the acute phase of SCI, and the effect of Tamoxifen on those changes using a rat model of traumatic SCI. Our hypothesis was that TMX could regulate the changes in gene expression after SCI. Furthermore, we performed a bioinformatic analysis to unravel the molecular pathways enriched during the acute phase of SCI with and without TMX treatment. Our results showed differential expression of genes related to the inflammatory response after SCI. Changes in gene expression of most of the genes involved in the inflammatory response were not affected after TMX treatment. Nevertheless, two relevant genes were downregulated after TMX treatment, *Ccr2* and *Mmp12*. In addition, we explored the spinal cord edema response to TMX by determining the amount of water content at the injury site.

## 2. Materials and Methods

### 2.1. Animal Model

Forty-four adult male, 269–365 g Long–Evans rats were used in this study. After surgery, all animals were housed in individual cages in a 12 h light/dark cycle under controlled temperature and humidity. A standard diet based on rat chow and water was provided ad libitum. Because TMX is a selective estrogen receptor modulator, we chose to work with male rather than female rats to avoid the competition for the receptor of TMX with endogenous female estrogens.

Experiments were performed in compliance with the local guidelines for the use and care of laboratory animals (NOM-062-ZOO-1999 Mexico) and the International Guide for the Care and Use of Laboratory Animals. All procedures were approved by the animal care committees at the National Institute of Rehabilitation of Mexico and the Institute “Proyecto CAMINA”. All efforts were made to minimize animal pain and discomfort and to reduce the number of animals used.

### 2.2. Study Groups

Animals were allocated to one of the two experiments as follows, one group (n = 24) for transcriptomic analysis and the second group (n = 20) for edema quantification.

For the transcriptome analysis, 24 animals were randomly assigned into four subgroups: (1) Non-injured without TMX administration (Sham/TMX-) (n = 6), (2) Non-injured with TMX administration (Sham/TMX+) (n = 6), (3) Injured without TMX administration (SCI/TMX-) (n = 6), and (4) Injured with TMX administration (SCI/TMX+) (n = 6). One sample from the Sham/TMX- group and one from the SCI/TMX- group was discarded due to low-quality RNA. Therefore, the groups were as follows: (1) Sham/TMX- (n = 5), (2) Sham/TMX+ (n = 6), (3) SCI/TMX- (n = 5), and (4) SCI/TMX+ (n = 6).

For the determination of tissue edema, 20 animals were randomly assigned into four subgroups: (1) Sham/TMX- (n = 5); (2) Sham/TMX+ (n = 5); (3) SCI/TMX- (n = 5); and (4) SCI/TMX+ (n = 5).

### 2.3. Injury and Postsurgical Care

Traumatic SCI was induced as previously described [21]. Briefly, animals were anesthetized with a mixture of ketamine (80 mg/kg) and xylazine (8 mg/kg) i.m. Next, a laminectomy was performed under aseptic conditions to expose the dorsal surface of the dural sac at the ninth thoracic (T9) level of the spine. The rats were positioned in the stereotactic frame by clamping the T8 and T10 spinous processes to inflict the injury. The SCI consisted of a lesion by contusion using the New York University weight-drop device (MASCIS impactor) by dropping a 10 g rod from a height of 50 mm, resulting in an injury of severe intensity. After causing the lesion, the wound was sutured in layers. Non-injured (sham) animals only underwent soft tissue surgery without laminectomy or SCI. Intramuscular tramadol (5 mg/kg) was administered perioperatively to prevent pain. Neurogenic bladder handling was performed by manual expression twice a day.

### 2.4. Neurological Assessment after SCI

Hind limb motor function was assessed as soon as the animals recovered from anesthesia using the locomotor rating scale from the Basso, Beattie, and Bresnahan open field test to ensure the effectiveness of the establishment of the injury [22]. The injury was considered successful when the animals showed flaccid bilateral paralysis of the hindlimbs and an absence of spontaneous movements and somatic reflexes at the tail.

### 2.5. Preparation and Administration of TMX

A suspension of TMX (T5648, Sigma-Aldrich, Saint Louis, MO, USA) was prepared by mixing each dose (5 mg/kg) in the vehicle (ethyl alcohol 100 μL + corn oil 1 mL). To achieve a homogenous mix, the suspension was prepared by heating the mixtures at 50 °C in a water bath for 5 min, followed by vortex agitation for 10 s three times. Rats received a single intraperitoneal dose of tamoxifen or vehicle 30 min after SCI or sham surgeries.

### 2.6. Collection and Processing of Spinal Cord Tissues

For transcriptome analysis, 24 h after spinal surgery, rats were euthanized with an overdose of intraperitoneal sodium pentobarbital (PISA, Mexico City, México) to collect spinal cord tissue. A 20 mm long spinal cord segment centered at the site of injury or equivalent in sham rats was removed and placed on a cooled plate, and then the dura matter was removed. Next, the spinal cord was sagittally divided into two similar segments, weighed, and cryopreserved at −80 °C until use.

For the determination of tissue edema, a segment of 1 cm of the spinal cord containing the lesion was weighed on an analytical scale. Next, tissues were placed on a hot plate (75 °C) for 4 h and weighed again. The tissue water content was calculated as follows:

WC = [(mwet − mdry)/mwet] × 100

where WC is the water content (in percentage of the mass), mwet is the mass of the fresh wet tissue, and mdry is the mass after the tissue was desiccated, as we also described previously [21].

### 2.7. RNA Extraction and Purification

Frozen tissues were homogenized using a Kinematica™ Polytron™ PT2500E desktop homogenizer (Fisher Scientific, Pittsburgh, PA, USA). Total RNA was extracted from homogenized tissues using the TRIzol (Invitrogen) method following the manufacturer’s recommendations. RNA concentration and integrity were obtained using the Agilent Bioanalyzer (Agilent Technologies, Santa Clara, CA, USA). Only samples with RNA Integrity Number (RIN) ≥ 7 were used for transcriptome analysis. 

### 2.8. Microarray Hybridization

Transcriptional profiles were analyzed using the Clariom S Assays Array (Thermo Fisher, Waltham, MA, USA) following the manufacturer’s instructions. Briefly, 200 ng of total RNA was converted to complementary DNA, labeled with the SensationPlus^TM^ FFPE Amplification and WT Labelling Kit (Affymetrix, Santa Clara, CA, USA), and hybridized on the array, which detects both mRNA and lncRNA. Arrays were washed, stained, and scanned using a Genechip Scanner 3000 7G (Affymetrix, Santa Clara, CA, USA). 

### 2.9. Bioinformatic Analysis

Microarray data were analyzed using R software version 4.1.0 [23] and Bioconductor version 3.13 [24]. Quality analysis was performed using the affycoretools package version 1.64 [25]. The data were normalized with the Robust Multichip Analysis (RMA) function of the oligo package version 1.56 [26], and for the differential analysis of gene expression, we used the limma package version 3.48.3 [27], using a linear model based on the Bayes empirical method [28]. Gene annotation was performed using the package pd.clariom.s.rat version 3.14.1 [29]. Representative data were considered significant if the *p* value was < 0.05, and log2FC > 0.7 or log2FC < −0.7 for TMX contrasts (SCI/TMX+ versus SCI/TMX- and Sham/TMX+ versus Sham/TMX-), and for SCI contrast (SCI/TMX- versus Sham/TMX- and, SCI/TMX+ versus Sham/TMX+) adjusted *p* value < 0.05, and log2FC > 1 or log2FC < −1.

The microarray data are available at the GEO database (https://www.ncbi.nlm.nih.gov/geo/, accessed on 5 September 2023), with the ID GSE229618.

### 2.10. Functional Enrichment Analysis

Functional enrichment analysis was performed using the g:GOSt tool through g:Profiler (https://biit.cs.ut.ee/gprofiler/gost 5 September 2023). This tool maps genes to known functional information sources and detects statistically significantly enriched terms [30]. The databases used for the enrichment analysis were WikiPathways (https://www.wikipathways.org/ 5 September 2023) [31], the Kyoto Encyclopedia of Genes and Genomes (https://www.genome.jp/kegg/ 5 September 2023) [32], and Reactome (https://reactome.org/ 5 September 2023) [33]. We also used GeneMANIA for the identification of interacting genes and the construction of networks [34].

### 2.11. Data Visualization

We generated the PCAs using ggplot2 version 3.3.6 [35], the volcano plots using EnhancedVolcano package version 1.10 [36], and the heatmaps using ComplexHeatmap package version 2.8.0 with hierarchical clustering and Euclidean distance as parameters. We additionally generated Venn diagrams using the VennDetail Shiny App (http://hurlab.med.und.edu:3838/VennDetail/ 5 September 2023) [37].

### 2.12. Statistical Analysis

For the analysis of edema, data were analyzed with multiple comparison two-way ANOVA followed by Tukey’s post hoc test. A *p* < 0.05 was considered significant.

## 3. Results

### 3.1. TMX Does Not Affect Acute Edema Formation Following Traumatic SCI

**Figure 1:**
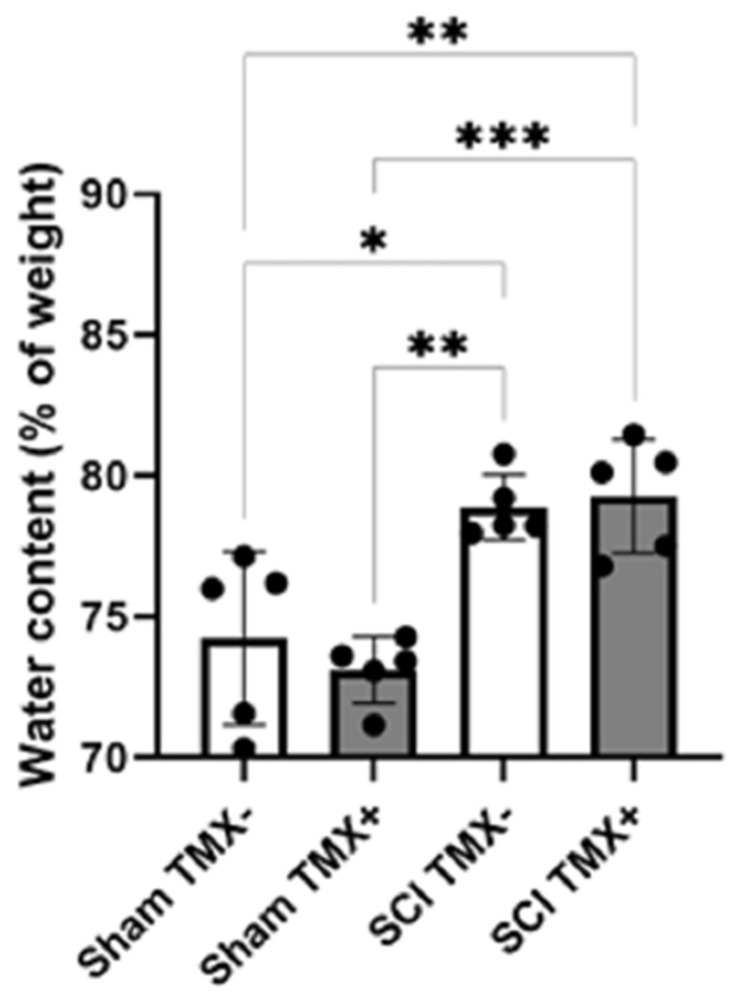
Edema formation in the spinal cord 24 h after traumatic SCI. The graph shows spinal cord water content measured 24 h after sham injury or SCI. No significant difference in the tissue water content was observed between the group injured without TMX (SCI TMX-) and the group injured treated with TMX (SCI TMX+). Sham TMX- was the non-injured and non-treated group. Sham TMX+ was non-injured and treated with TMX. Data are expressed as the mean ± SD (n = 5 per group, total N = 20). Significant differences were only observed in the comparison between the non-injured versus injured groups. * *p* < 0.05. ** *p* < 0.01. *** *p* ≤ 0.001. Two-way ANOVA followed by Tukey’s post hoc test was performed for this analysis.

We measured vasogenic edema formation to investigate whether the early administration of TMX could modify vasogenic edema formation in the first 24 h after contusion, which is considered the critical period. For this purpose, we determined the net weight of the wet spinal tissue (10 mm long). Next, the spinal segment was heat-dehydrated for 4 h, after which the net weight of dry tissue was determined again. According to the formula described in the methods section, the content of water in the tissue was calculated. Figure 1 shows that there was an increase in the percentage of water after 24 h of traumatic SCI independent of TMX treatment compared to the sham/TMX- group (two-way ANOVA, F = 15.77, DF 3,12, *p* = 0.0002). Nevertheless, there was no statistically significant difference in the percentage of water content between the SCI/TMX- and SCI/TMX+ groups (Figure 1). Therefore, TMX does not affect vasogenic edema formation in the first 24 h after injury.

### 3.2. Differential Gene Expression Analysis

**Figure 2:**
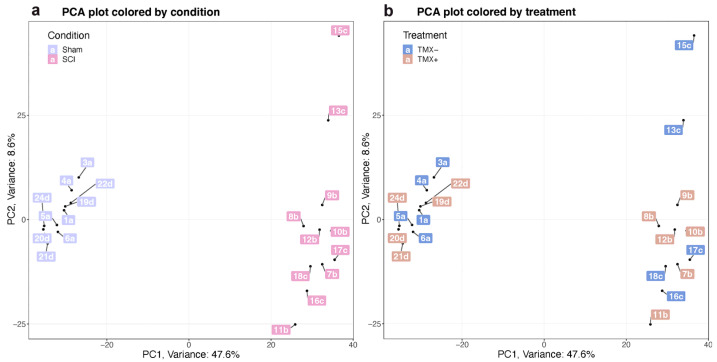
Principal component analysis (PCA) of microarray expression data. (**a**) PCA plot colored by condition, sham in lilac, SCI in pink. (**b**) PCA plot colored by treatment, without tamoxifen (TMX) in blue, treated with TMX in salmon. The number of each sample corresponds to the ID given for experimental purposes.

Gene expression was analyzed using the Clariom S rat Assays Microarray, as mentioned in the methods section. Rats were distributed into four groups: (1) Sham/TMX-, (2) Sham/TMX+, (3) SCI/TMX-, and (4) SCI/TMX+. Through principal component analysis (PCA) of the expression profiles, we grouped the samples according to their variation. Based on the contrast by condition (SCI versus sham), samples were classified into their respective groups. However, substantial sample-to-sample variance was found for the contrast treatment (TMX+ versus TMX-). Therefore, samples could not be easily grouped based on this last criterion (Figure 2).

**Figure 3:**
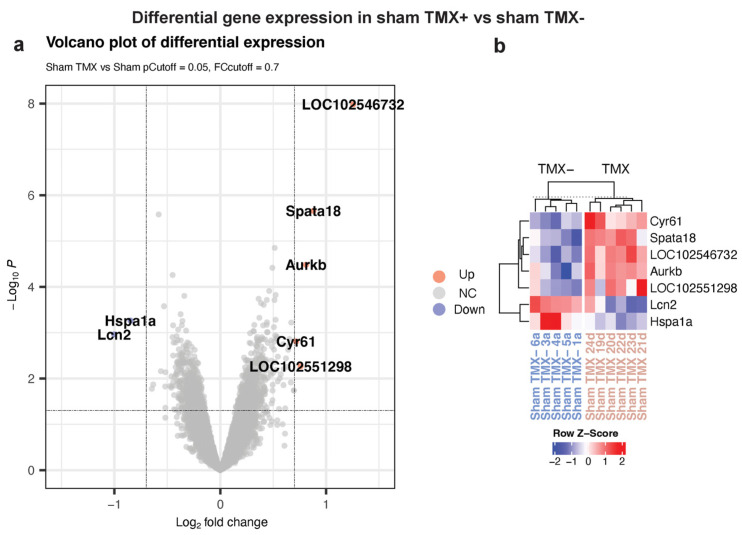
Differentially expressed genes in sham/TMX+ versus sham/TMX- rats. Volcano plot (**a**) and heatmap (**b**) showing the responsive genes to TMX treatment in sham rats.

To investigate whether there was an effect of TMX on sham rats, we compared expression data from the sham/TMX- group versus the sham/TMX+ group using the limma package. From this comparison, seven transcripts showed differential expression, suggesting a discrete effect of TMX on gene expression in the spinal cord (Figure 3a). A heatmap using these seven genes grouped all the individuals of each condition (Figure 3b). The genes that responded to tamoxifen were Ccn1, cellular communication network factor 1; Lcn2, Lipocalin 2; Aurkb, aurora kinase B; Spata18, spermatogenesis associated 18; Hspa1a, heat shock protein family A (Hsp70) member 1; LOC102551298, an MLV-related proviral Env polyprotein-like; and the uncharacterized LOC102546732 (Table 1).

**Figure 4:**
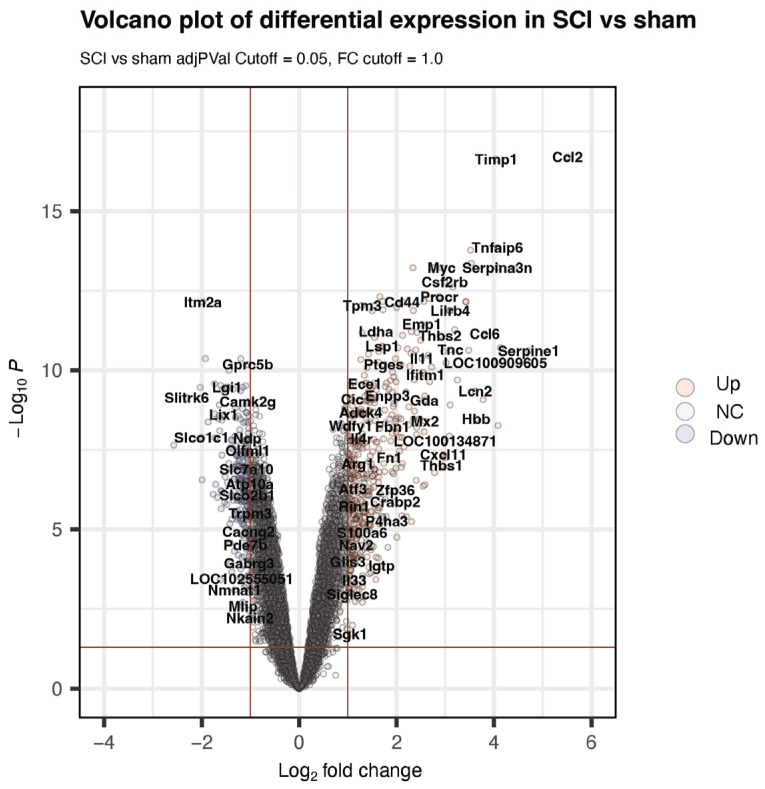
Volcano plot showing the responsive genes to spinal cord injury without TMX treatment. Genes differentially expressed when comparing the group with spinal cord injury (SCI) versus sham. Upregulated genes are shown in red, and downregulated genes are shown in blue. Table 2. Enrichment pathway analysis. Top five enriched functions when comparing the SCI/TMX- versus sham/TMX- groups.

To reveal the gene expression changes secondary to the effect of SCI, we compared the Sham/TMX- and SCI/TMX- groups (Figure 4). A total of 708 transcripts showed differential expression (Supplement Table S1). From them, 249 genes were downregulated, and 459 were upregulated. To investigate the cellular functions enriched in response to SCI, we used the 708 differentially expressed genes as input for enrichment analysis in gProfiler (Table 2). Enrichment analysis using the database WikiPathways returned the spinal cord injury (WP2433), as the top enriched function. Interestingly, the other four enriched functions are related to the inflammatory response. We also looked for the enriched pathways using KEGG and Reactome. In both databases, the top five enriched functions included pathways related to the inflammatory response. Of the 708 differentially expressed genes, 102 participate in one or more of the inflammatory response pathways enriched (Supplement Table S2). These results agree with the relevance of the activation of the inflammatory response during the acute phase of spinal cord injury, as previously established. 

**Figure 5:**
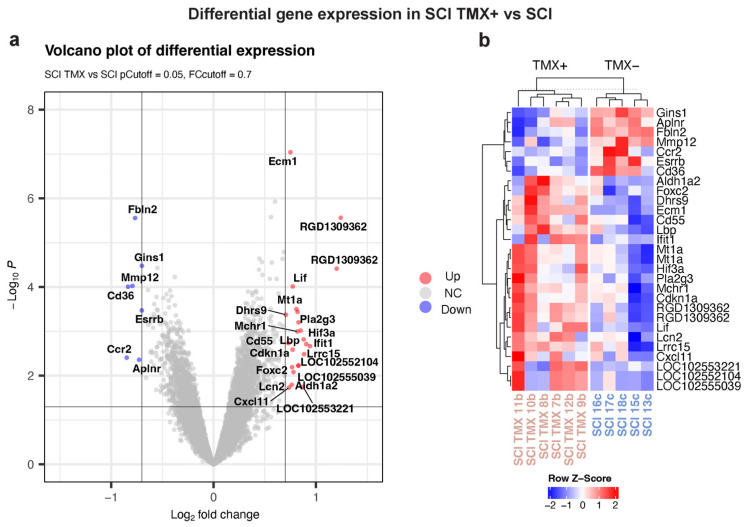
Responsive genes to TMX in the groups of rats with spinal cord injury after treatment with (TMX). Genes with differential expression when comparing the group with SCI/TMX+ versus SCI/TMX-. Volcano plot (**a**) and heatmap (**b**) showing the responsive genes to TMX treatment.

From the comparison between the Sham/TMX- and SCI/TMX- groups, we acknowledged a genetic response induced by SCI on rats. For determining the effect of TMX on the gene expression response toward SCI, our subsequent comparison was between the SCI/TMX- and SCI/TMX+ groups. This comparison revealed only 30 transcripts with differential expression, and through unsupervised hierarchical clustering, these 30 transcripts grouped all the individuals from each condition (Figure 5 and Supplement Table S3). For a better understanding of the changes induced by TMX on injured rats, we used these 30 transcripts to perform enrichment analysis in gProfiler using the same three databases. However, no enriched pathways were retrieved. As described above, 708 genes were differentially expressed when comparing Sham/TMX- versus SCI/TMX-. From these 708 genes, 102 genes participate in one or more of the inflammatory response pathways showing enrichment. The independent comparison of the SCI/TMX- group versus the SCI/TMX+ group revealed a set of 30 transcripts with differential expression. The intersection between these two sets of differentially expressed genes comprised 14 genes, presented in italics in Supplement Table S3. We looked for those genes belonging to the enriched inflammatory pathways within these 14 genes in the intersection. From these 14 genes in the intersection, Mmp12 was upregulated in response to SCI. Nevertheless, when TMX was administered, it changed to downregulation (See Supplement Tables S1 and S3). Additionally, we found only five genes participating in the inflammatory response pathways, within the 14 genes in the intersection, these five genes are highlighted in blue text in Supplement Table S3. Four out of these five genes were overexpressed in both comparisons, among them, the case of Lcn2 seems interesting. In sham rats, TMX seems to downregulate the expression of Lcn2, as shown in Table 1 and Figure 3. After SCI, Lcn2 showed overexpression, this overexpression remained even after the treatment with TMX. This finding suggests that TMX was unable to counteract the effect of SCI on Lcn2 expression, or it is not possible to rule out a synergistic effect of TMX on the expression of certain genes. The fifth gene, Ccr2, changed to downregulated in the SCI/TMX- versus SCI/TMX+ comparison. Ccr2 is the main chemokine receptor of the monocyte/macrophage linage, it changed from overexpressed after SCI (Log2FC 1.58, see Supplement Table S1), to downregulated (Log2FC −0.84, see Supplement Table S3) after TMX treatment. This comparison allowed us to specify a differential expression due to a regular inflammatory response after a SCI and the expression after the use of tamoxifen showed with the other groups. 

**Figure 6:**
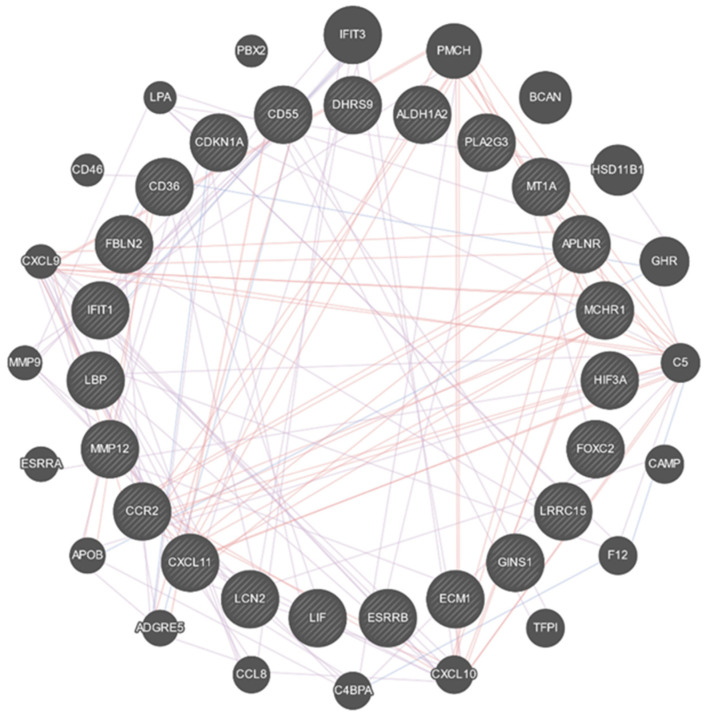
Interaction network between the transcripts with differential expression between the SCI/TMX- and SCI/TMX+ groups. In the inner circle are the 22 annotated genes with differential expression from the comparison between the SCI/TMX- and SCI/TMX+ groups. The outer circle is composed of the 21 genes able to interact with the genes from the inner circle according to Genemania. Interaction can be assigned based on evidence of co-expression (purple lines), colocalization (blue lines), or physical interaction (red lines).

To better understand the possible involvement of these 30 transcripts in the response to TMX after SCI, we built an interaction network between them. For this purpose, we selected only the annotated genes from these 30, and we found annotations for 22 of them. Afterward, we looked for genes with a known interaction with these 22 genes of interest using Genemania. The analysis of the interaction with Genemania revealed 21 genes able to interact with the 22 differentially expressed annotated genes. A total of 43 genes were considered for the interacting network (Figure 6). 

We further performed an enrichment analysis in gProfiler using these 43 genes as input. Table 3 depicts the enriched pathways according to the same three databases used in the previous enrichment analysis. Interestingly, the spinal cord injury pathways remained significantly enriched, although it was not the first one. From this analysis, we can observe that some inflammatory-response pathways, such as Cytokine–cytokine receptor interaction and the Toll-like receptor signaling pathway remained enriched. Further experiments need to be conducted with two purposes. First to determine if the pathways showing enrichment after TMX treatment could be related to the effect of TMX; second, to investigate the mechanisms by which TMX regulates the pathways no longer overrepresented after its administration to injured rats.

To further investigate the effect of TMX on the genetic response of the rats toward SCI, we compared the sham/TMX+ and SCI/TMX+ groups. From this comparison, we observed 706 transcripts with differential expression. When we intersected these transcripts with the 708 differentially expressed transcripts derived from the comparison of the SCI/TMX- versus sham/TMX- groups, 522 were in the intersection (Supplement Figure S1A). We conducted an enrichment analysis using these 522 transcripts using the above-mentioned databases (Supplement Table S1). Interestingly, most of the enriched pathways retrieved were also observed in the enrichment analysis shown in Table 2. This observation suggests a relevant involvement of these 522 transcripts in the genetic response toward SCI, and TMX does not seem to have an effect on them. The remaining 184 transcripts from the sham/TMX+ versus SCI/TMX+ comparison were also used as input for enrichment analysis. However, only two pathways were retrieved, the Nicotine addiction (rno05033) and the GABA A receptor activation (R-RNO-977441) pathways from KEGG and Reactome, respectively. Our final comparison comprised the sham/TMX- versus SCI/TMX+ groups. From this comparison, 619 transcripts showed differential expression. In this last comparison, we observed the effect of both factors, SCI and TMX, on the rats; therefore, it is complicated to discriminate which genes correspond to each factor. Supplement Figure S1B summarizes the findings regarding the transcripts showing differential expression between the different comparisons.

## 4. Discussion

Traumatic SCI has become a public health issue worldwide, with an incidence ranging from 13 to 163.4 per million in developed and undeveloped countries, respectively [38]. Despite the implementation of new therapeutic strategies, fatal outcomes can occur in up to 22% and 20% of cases in developed and undeveloped countries, respectively [38]. Unraveling the cellular processes occurring during the acute phase of SCI could provide valuable knowledge for developing new therapeutic strategies. 

Here, we used a rat model of traumatic SCI to identify the changes in gene expression during the acute phase through transcriptome analysis. Because TMX is a selective estrogen receptor modulator, we chose to work with male rather than female rats to avoid the competition for the receptor of the drug with endogenous female estrogens. Although we did not find that TMX alleviated tissue edema 24 h after SCI, the molecular characterization of TMX-induced changes in gene expression, showed that the differential expression of some inflammatory genes, induced by SCI, changed after TMX treatment.

### 4.1. Transcriptome Changes Induced by SCI

We identified the genes with differential expression between rats with SCI and without SCI in the absence of TMX. This analysis revealed the presence of 708 differentially expressed genes. Enrichment analysis with these 708 genes as input retrieved the pathway of spinal cord injury as the most significant, according to the WikiPathways database. A total of 26 genes from the input belonged to this pathway. These genes are mainly involved in the activation of the monocyte/macrophage response. This response has been the subject of scrutiny because proinflammatory macrophages are more abundant during the acute phase of SCI, and during the subacute phase, they are more abundant in the presence of macrophages involved in immunomodulation and tissue repair [39]. Interestingly, we found differentially expressed genes previously identified as key players in macrophage activation, such as *Arg1*, *Nos2*, *Il6*, and *Il1b*. Drugs modifying the expression of these genes are promising resources for treating SCI [40]. 

### 4.2. Effect of TMX on Transcriptome Changes Caused by SCI

When we treated the injured rats with TMX, we only observed 30 differentially expressed genes compared to the injured rats with no TMX. The spinal cord injury pathway was no longer retrieved using these 30 genes as input for enrichment analysis in WikiPathways. In fact, no pathways were significantly enriched. Of the 30 differentially expressed genes, only *Ccr2* and *Mmp12* belonged to the spinal cord injury pathway. It is important to note that both genes changed their differential expression from upregulated in the absence of TMX treatment to downregulated when TMX was administered. There is evidence demonstrating an important upregulation of *Mmp12* after SCI in rodent models. Interestingly, *Mmp12* has been considered as a potential therapeutic target for SCI treatment [41]. Further experiments are needed to corroborate the effect of TMX on Mmp12 expression at different time points after SCI. Based on this observation, TMX treatment might be a factor modulating some genes involved in the genetic response associated with spinal cord trauma. 

### 4.3. TMX as Modulator of the Inflammatory Response after SCI

Tamoxifen has been proven to have a positive effect during the acute and subacute phases of SCI [42,43]. Experiments using different rat models have corroborated that TMX effectively modulates the immune response during the first 24 h after SCI [44]. TMX reduces the recruitment and activation of inflammatory cells at the site of injury. This effect seems to be due to the partial inhibition of the IKK/NF-kB signaling pathway and the expression of inflammatory factors, such as IL-6 [20,45]. Intriguingly, it seems that this response is not estrogen-receptor-mediated. Our results agree with these observations since we observed overexpression of the Il-6 gene in the absence of TMX. There are only a few analyses of the transcriptome in rodent models of SCI, either in mice [46] or rats [47,48], and there is also a bioinformatic analysis using transcriptome data from mice retrieved from a public repository [49]. Those studies also observed enrichment of inflammation and immune response in the acute phase of the injury [46,47,48,49]. A previous study measured the effect of methylprednisolone on gene expression at different time points. The best response to treatment was observed in the first 8 h after injury, responsive genes were also involved in the inflammatory response [8]. This observation agrees with our results pointing out the importance of SCI pharmacological treatment within the first 24 h. This time window prevents the immune response from exacerbating, leading to neuronal damage. The other study identified unique differentially expressed genes at different time points. They found *Ccr1* and *Nos2* to be uniquely differentially expressed 24 h after SCI [47]. We also observed overexpression of these two genes after SCI; however, treatment with TMX does not seem to have an effect on the overexpression of these two genes. Of particular interest is the case of *Lcn2*, TMX was able to downregulate its expression in non-injured rats. In rats with SCI, *Lcn2* was found overexpressed, this overexpression was persistent even in the presence of TMX. This observation pointed out that TMX could not be effective in counteracting the effect of SCI, at this time point, on certain genes involved in inflammatory reaction. On the other hand, TMX effectively suppressed the overexpression of *Ccr2*, which is a key factor for monocyte/macrophage recruitment at injured zones [44].

Based on these observations, TMX seems to have a mild effect on the expression of key genes participating in the inflammatory response. In addition, the enrichment analysis using KEGG showed that inflammatory pathways were still present after TMX treatment.

### 4.4. Transcriptome Analysis for Identifying Potential Biomarkers of SCI

Previous studies conducting transcriptome analysis have not achieved the full delineation of the activated pathways after SCI but have provided valuable insights into potential biomarkers related to injury severity and functional recovery. For example, a previous study employing a system-analysis approach identified the product of the gene Anxa1 as a promising indicator of the severity of injury. The evidence showed that the levels of the Anxa1 protein could discriminate between moderate and severe injury with an accuracy of 93% [50]. In our study, all rats with SCI presented severe injury. Anxa1 was found to be upregulated after SCI, and treatment with TMX reversed Anxa1 overexpression. This observation suggests that TMX might prevent Anxa1 overexpression, supporting a protective role of TMX within the first 24 h after SCI. Nevertheless, experiments treating rats with severe and moderate injuries are necessary to corroborate the role of TMX. 

This study contributes to the current knowledge regarding the changes in gene expression after SCI using a rat model. Our results showed differential expression of genes participating in the inflammatory response during the acute phase of SCI. TMX does not have the side effects of glucocorticoid drugs such as methylprednisolone, therefore, its value as a therapeutic resource deserves more investigation. Some limitations of our study include a small number of animals per group. However, our results on gene expression agree with previously published data. Additionally, we only analyzed SCI at one time point during the acute phase. Nevertheless, the current evidence stresses that this is the critical window for treating affected individuals. We do not have injuries of different severities; therefore, we do not have experimental support for the potential role of *Anxa1* as a useful biomarker. Nevertheless, the fact that *Anxa1* was overexpressed after injury opens the possibility that it is a potential biomarker. Our study is limited to gene expression analysis, further analyses of protein expression would corroborate our findings. However, our gene expression results agree with previous data, and protein analysis was beyond the scope of this study. We did not consider another route for TMX administration, making it difficult to extrapolate our results to a clinical scenario. However, we used one of the most accepted routes for drug administration in rat models. The data presented here add to the body of evidence about the critical role of the inflammatory reaction on SCI outcome. This and other gene expression profiles of SCI would be useful for selecting the best therapeutic targets and biomarkers in the future. Furthermore, our study contributes knowledge of the potential use of TMX for treating SCI. Based on our and other studies, the translation of TMX to the clinic requires additional investigation in animal models for testing its efficacy at different time points and on injuries of different severity. 

## 5. Conclusions

Cases of SCI are expected to increase in all countries, and new therapeutic resources are needed to improve the quality of life of affected individuals. The data presented here show that TMX has a mild effect on the genetic inflammatory response during the acute phase of SCI. These results contribute data regarding the effect on gene expression of TMX during the acute phase of SCI in a rat model. 

## Figures and Tables

**Table 1:** Differentially expressed genes from the comparison between the sham TMX+ and sham TMX- groups.

Transcript ID	Gene Symbol	Gene Name	Log2FC	*p* Value
mRNARGD7697195_1	*LOC102546732*	Uncharacterized LOC102546732	1.25611	1.05 × 10^−8^
NM_199374	*Spata18*	Spermatogenesis associated 18	0.8805877	2.20 × 10^−6^
NM_053749	*Aurkb*	Aurora kinase B	0.8106	3.29 × 10^−5^
ENSRNOT00000061950	*Hspa1a*	Heat shock 70 kD protein 1A	−0.8484825	0.0005
NM_130741	*Lcn2*	Lipocalin 2	−1.0030	0.0010
NM_031327	*Cyr61*	Cysteine-rich, angiogenic inducer, 61	0.7176	0.0015
mRNARGD7732176_1	*LOC102551298*	MLV-related proviral Env polyprotein-like	0.7595	0.0054

Log2FC, Expression fold change expressed in logarithm base 2.

**Table 2:** Enrichment pathway analysis. Top five enriched functions when comparing the SCI/TMX- versus sham/TMX- groups.

Database	Pathway	ID	Adjusted *p* Value
**WP**	Spinal cord injury	WP2433	0.00002
	Burn wound healing	WP5057	0.00153
	Type II interferon signaling (IFNG)	WP1289	0.00555
	Prostaglandin synthesis and regulation	WP303	0.01417
	Toll-like receptor signaling pathway	WP1309	0.04706
**KEGG**	Malaria	KEGG:05144	6.3 × 10^−7^
	TNF signaling pathway	KEGG:04668	6.3 × 10^−7^
	IL-17 signaling pathway	KEGG:04657	1.5752 × 10^−6^
	Cytokine-cytokine receptor interaction	KEGG:04060	8.8134 × 10^−6^
	Viral protein interaction with cytokine and cytokine receptor	KEGG:04061	8.8134 × 10^−6^
**Reactome**	Neutrophil degranulation	R-RNO-6798695	0.00002
	Innate Immune System	R-RNO-168249	0.00189
	Class A/1 (Rhodopsin-like receptors)	R-RNO-373076	0.00858
	Chemokine receptors bind chemokines	R-RNO-380108	0.01344
	Hemostasis	R-RNO-109582	0.01358

WP, WikiPathways. KEGG, Kyoto Encyclopedia of Genes and Genomes.

**Table 3:** Enrichment pathway analysis. Top enriched functions comparing the SCI/TMX- and SCI/TMX+ groups.

Database	Pathway	ID	Adjusted *p* Value
* WP	Complement and coagulation cascades	WP547	0.02669
	Spinal cord injury	WP2433	0.04960
	Toll-like receptor signaling pathway	WP1309	0.04960
**KEGG**	Complement and coagulation cascades	KEGG:04610	1.6415 × 10^−05^
	Viral protein interaction with cytokine and cytokine receptor	KEGG:04061	0.00392
	Cytokine-cytokine receptor interaction	KEGG:04060	0.00392
	Toll-like receptor signaling pathway	KEGG:04620	0.00392
	Fat digestion and absorption	KEGG:04975	0.00630
**Reactome**	Peptide ligand-binding receptors	R-RNO-375276	0.00014
	G alpha (i) signaling events	R-RNO-418594	0.00014
	GPCR ligand binding	R-RNO-500792	0.00014
	Class A/1 (Rhodopsin-like receptors)	R-RNO-373076	0.00235
	Signaling by GPCR	R-RNO-372790	0.00625

WP, WikiPathways. KEGG, Kyoto Encyclopedia of Genes and Genomes. * Only three pathways were retrieved when WP was used as the source database.

## Data Availability

The microarray data are available at the GEO database (https://www.ncbi.nlm.nih.gov/geo/, accessed on 5 September 2023), with the ID GSE229618.

## Supplementary Materials

The following supporting information can be downloaded at: https://www.mdpi.com/article/10.3390/cimb45090472/s1, Table S1: DEG_SCI TMX- vs. SHAM TMX-; Table S2: Genes participating in Enriched pathways; Table S3: DEG_SCI TMX- vs. SCI TMX+; Figure S1: (A) Venn diagram. (B) Summary of the number of differentially expressed genes.
