# Supplementary material for: Administration of Tamoxifen Can Regulate Changes in Gene Expression during the Acute Phase of Traumatic Spinal Cord Injury
Source: Curr Issues Mol Biol. 2023 Sep 13;45(9):7476–91. doi: 10.3390/cimb45090472 (PMC10529143; doi:10.3390/cimb45090472)
Supplement: Supplementary file 1 [file cimb-45-00472-s001.zip › cimb-2562841-supplementary.pdf]

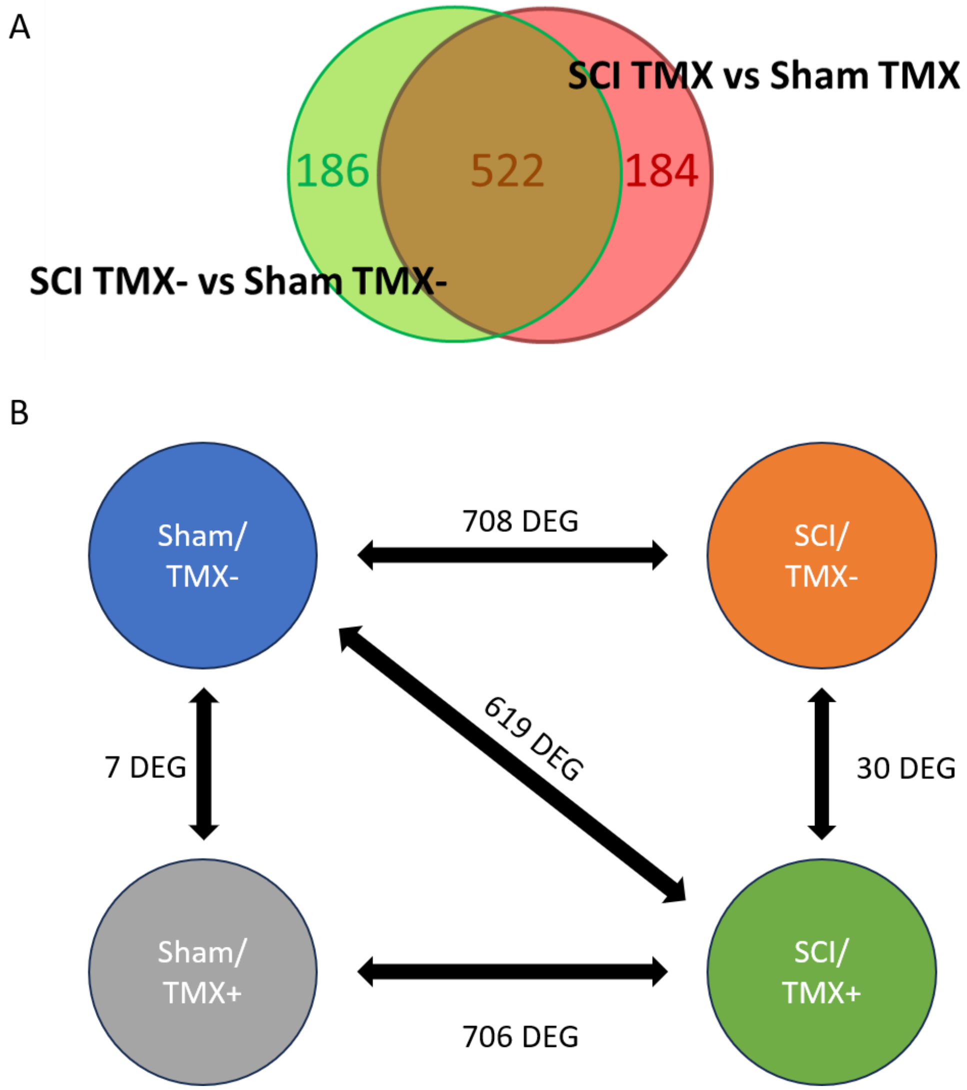

Figure S1. (A) Venn diagram showing that 522 differentially expressed genes comprised the intersection of two comparisons, SCI/TMX- versus sham/TMX- and SCI/TMX+ versus sham/TMX+. (B) Summary of the number of differentially expressed genes (DEG) observed in each comparison shown.
